# Joint User Clustering and Graph Coloring Based Pilot Assignment for Cell-Free Massive MIMO Systems

**DOI:** 10.3390/s23115014

**Published:** 2023-05-23

**Authors:** Xinyu Huang, Yubo Wang, Shiyong Chen, Yan Li, Yucheng Wu

**Affiliations:** 1 School of Microelectronics and Communication Engineering, Chongqing University, Chongqing 400044, China; 2 Beijing Smart-Chip Microelectronics Technology Co., Ltd., Beijing 100192, China

**Keywords:** cell-free massive MIMO, pilot contamination, AP selection, user clustering, graph coloring

## Abstract

Pilot contamination due to pilot reuse seriously mitigates the performance of the cell-free massive multiple-input multiple-output (MIMO) systems. In this paper, we propose a joint pilot assignment scheme that employs user clustering and graph coloring (UC-GC) to reduce pilot contamination. The proposed method consists of two steps: firstly, we utilize AP selection to classify all users; secondly, we assign pilots to users with more severe pilot contamination using the graph coloring algorithm and then assign pilots to the remaining users. The numerical simulation results show that the proposed scheme outperforms existing pilot assignment schemes and significantly improves throughout with low complexity.

## 1. Introduction

Cell-free massive multiple-input multiple-output (MIMO) technology presents itself as a promising solution for meeting the expectations of the increasing numbers of wireless users and the high-speed demands beyond 5G networks. It combines the advantages of massive MIMO and distributed antenna technologies, with the deployment of numerous access points (APs) equipped with single or multiple antennas across the communication area. These APs provide services for randomly distributed users within the area via the same time/frequency resource and are linked to the central processing unit (CPU) through backhaul links to provide coherent cooperation [1,2]. The user is surrounded by adjacent APs in the cell-free scheme, effectively shortening the average distance between the AP and the user and significantly reducing path loss. Consequently, this eliminates the traditional notion that the user at the edge of the cell experiences the worst performance [3,4,5]. Moreover, there is no need for cooperation between APs, so there is no additional pilot resource overhead or complex precoding design and scheduling issues. Compared with the traditional cellular network, the cell-free network system obtains more macro diversity gain, higher coverage probability, and greater spatial multiplexing through favorable propagation conditions [6,7]. Due to its significant performance advantages, it is considered to be one of the most promising key technologies in future mobile communication systems [8].

Although it is possible to deploy a cell-free system regarding the architecture of a cloud radio access network (C-RAN), due to the AP deployment, the CF paradigm itself, and the signal processing inherited from massive MIMO, is substantially different from C-RAN [9,10]. In a cell-free network, similar to a multi-cell network, channel estimation is completed during the uplink pilot training phase. The APs use the channel state information (CSI) acquired to decode uplink data transmission and to precoding downlink data transmission and instantaneous channel state information is not shared between the APs [11]. In the uplink pilot training phase, the ideal state is to assign mutually orthogonal pilots to all users in order to obtain accurate CSI. However, if the coherence interval duration is short or the number of users in the system is large, the finite number of pilots cannot maintain orthogonality between pilots assigned to all users. This inevitably results in pilot multiplexing, which negatively affects channel estimation, brings pilot contamination, and affects system performance [12].

As the number of APs and users in the cell-free network increases, the channels between them are typically orthogonal, leading to a significant performance improvement compared with cellular networks. However, due to the coherence time limitation, the system inevitably suffers from pilot contamination, which hinders further performance improvement. Therefore, developing a reasonable and efficient pilot assignment scheme can effectively alleviate the interference between users, thereby enhancing the overall system performance.

## 2. Related Works

By enabling macrodiversity and effectively utilizing limited power/bandwidth resources, a scheme where each user selects a portion of APs to serve them can outperform a fully connected scheme where all APs serve all users [13,14]. Thus, to reduce pilot contamination and improve system performance, the AP selection algorithm should be designed first.

In [15], the authors proposed two AP selection algorithms, one based on the maximum large-scale fading and the other based on the received signal power. The AP selection algorithm based on the maximum large-scale fading ranks the large-scale fading coefficients between the users and all APs in descending order, then selects an AP set. The criterion for the selection is that the sum of the large-scale fading coefficients must surpass a certain threshold to provide service to the user. This method has low computational complexity. The other AP selection algorithm that is based on the received signal power selects an AP by utilizing the power control coefficient. This approach requires power optimization before AP selection, which may lead to high computational complexity. In [16], a joint user clustering and AP selection scheme were proposed. This scheme initially leveraged a hierarchical clustering algorithm to group users with high channel similarity into the same cluster. Afterward, the scheme combined the optimized power control coefficient and the channel estimation error to propose a suboptimal AP selection scheme. The results indicated that the combined user clustering and AP selection scheme could reduce the backhaul link pressure of the system. However, the scheme only slightly enhanced the system’s performance. The authors of [17] studied the AP selection problem from the perspective of game theory. They modeled the formation of user-centered AP service clusters as a local altruistic game. Simulations confirmed both the convergence of the proposed algorithm and its superior performance compared with other methods. In [18], the authors proposed an AP selection algorithm that was based on clusters and employed machine learning to minimize the issues of time complexity and pilot contamination. In [19], the authors deduced the channel gain between all users and all APs, calculated a reference value for each user’s channel quality, and used this reference value to select APs. A large number of simulations have shown that this algorithm can improve system performance.

If each user is randomly assigned with a pilot, this method can result in severe pilot contamination, as long as two users connected to the same AP reuse the same pilot [11]. Consequently, significant research has been dedicated in recent years to designing pilot assignment schemes that aim to further alleviate the issue of pilot contamination.

Different pilot allocation schemes have been proposed in the literature to improve system performance and fairness in wireless communication networks. In [11], a greedy algorithm was applied to assigning pilots by updating the pilot of users with the lowest uplink rate iteratively in order to improve system fairness. However, this scheme only improved the performance of the worst users and did not significantly enhance overall system efficiency. Similarly, the authors of [20] proposed a greedy pilot assignment scheme based on location information. However, compared with theoretical results, this scheme could only achieve limited performance enhancement. In [21], a pilot assignment scheme based on a genetic algorithm was proposed to maximize the average downlink achievable rate. The numerical results showed that the scheme based on a genetic algorithm was superior to other traditional schemes. However, genetic algorithms lacked local search capability and were prone to “premature” convergence. The authors of [22] proposed a pilot allocation scheme based on a tabu search. This iterative algorithm was designed to avoid local optima results and reduce pilot contamination. The authors in [23] refined the solution space of the pilot allocation algorithm proposed in [22] and improved its performance by incorporating large-scale fading coefficients to find an optimal pilot allocation scheme with low complexity. Moreover, the authors in [24] proposed a joint uplink and downlink pilot allocation algorithm to minimize the network’s utility function. They designed the scheme to consider both uplink and downlink pilot contamination to ensure uniform service quality in the entire network. The authors of [25] utilized a heap-based algorithm to reduce pilot pollution and improve spectral efficiency. The authors in [26] introduced a pilot assignment scheme that employed the Hungarian algorithm to enhance system throughput and fairness. The authors solved an optimization problem by fusing the genetic algorithm and the Hungarian algorithm [27]. In [28,29,30], the authors established an interference graph according to pilot assignment conflicts among users and proposed a pilot assignment scheme based on graph coloring. In [31], the authors suggested a distance-based pilot allocation scheme that chose the same pilot for two users with a sufficient distance between them. In [32], the design of massive MIMO non-orthogonal multiple access (NOMA) systems was investigated; the users were grouped into spatial-clusters and a null-space based precoder was used to cancel the inter-cluster interference. However, such null-space based precoding can be prohibitively complicated for massive MIMO. In [33], the authors investigated the implementation of a power-domain NOMA technique in cell-free massive MIMO systems. Simulations confirmed that NOMA was capable of serving twice the number of users as orthogonal multiple access (OMA) and achieved a greater sum rate when serving a great number of users. Nevertheless, in scenarios where the number of users was low, OMA performed better than NOMA, owing to intra-cluster pilot contamination and imperfect successive interference cancellation (SIC) techniques. In [34], the authors proposed a pilot assignment algorithm based on K-means clustering, which divided users into different clusters to avoid pilot contamination caused by pilot reuse between users from a spatial perspective. However, this scheme did not consider the interference between users among the different clusters. Most existing schemes aim to avoid reusing the same pilot among adjacent users and only optimize pilot assignment based on the geographical location between users. This scheme was not accurate enough.

## 3. Contribution

In traditional cellular systems, pilot assignment is performed by allocating a set of predefined pilot symbols to each cell. However, in cell-free massive MIMO systems, which lack physical cell boundaries, the APs and users are randomly distributed throughout the network. Consequently, the design of the pilot assignment process in cell-free massive MIMO systems must consider the spatial distribution of the APs and users. One of the methods used for pilot assignment in cell-free massive MIMO systems is to use a clustering algorithm to group APs and users according to the spatial proximity criterion and then reuse an orthogonal set of pilot signals per cluster to avoid intra-cluster interference. User clustering groups users with similar characteristics into groups and then utilizes the same set of pilot resources to serve all users in a group. This technique reduces interference among users and improves overall system performance. However, considering only the pilot assignment within each user cluster may result in serious inter-cluster user interference. Therefore, we take into account the clustering results in the design of the pilot assignment scheme.

We propose an effective pilot assignment scheme aiming to mitigate pilot contamination in cell-free massive MIMO systems. The specific contributions of this paper are outlined below:

(1) Considering the effects of channel estimation error, power control, and non-orthogonal pilots, we derive a closed-form expression for the achievable uplink and downlink rates of users having a finite number of APs. The proposed strategies utilize the knowledge of the large-scale fading coefficients as a stand-in for the true distances between the users and the APs. The strategy involves selecting a specific set of APs for each user based on their respective large-scale fading coefficient values.
(2) To effectively utilize the limited number of mutually orthogonal pilots and reduce pilot contamination from a spatial perspective, users connected to the same APs are grouped based on the users’ AP selection result, while limiting the number of users in each group to the number of orthogonal pilots to prevent the reuse of only a few pilots.
(3) An effective scheme for reducing pilot contamination is proposed. The scheme first assigns pilots to inter-cluster users who are connected to the same APs but belong to different clusters based on the clustering results and then to intra-cluster users who are connected to the same APs and belong to the same cluster. The problem of pilot assignment for inter-cluster users is mapped to a graph-coloring problem and a metric with high accuracy is developed to measure inter-cluster users’ potential interference intensity. Finally, dynamic pilot assignment is implemented for all inter-cluster users and orthogonal pilots are assigned within each cluster.
(4) Numerous simulation results confirm that the proposed scheme is effective in reducing pilot contamination and significantly improving system performance.

The remainder of this paper is structured as follows. Section 4 provides a brief description of the system model and examines the sources of pilot contamination. Section 5 outlines the specific details of the proposed scheme in this paper. In Section 6, we present the simulation results and engage in discussion. Finally, our conclusions are presented in Section 7.

## 4. System Model

**Figure 1:**
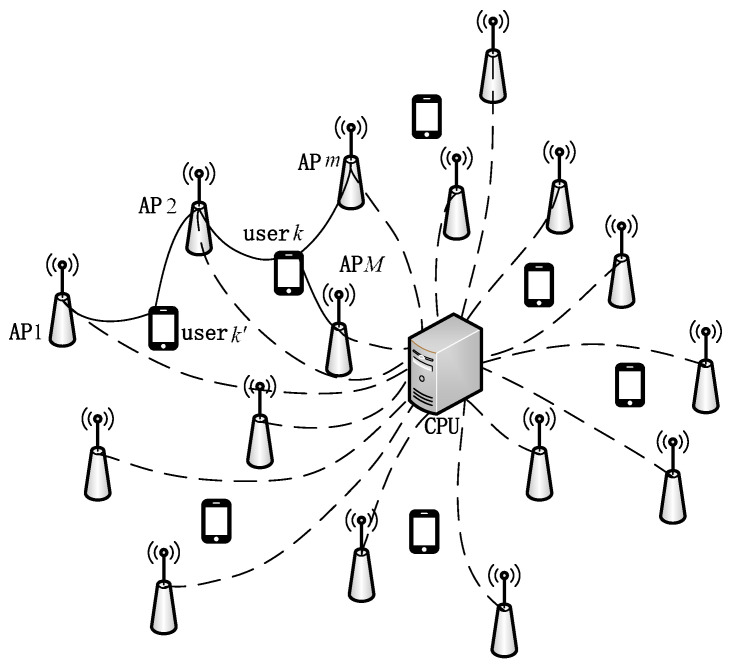
Cell-free massive MIMO network.

We consider the cell-free massive MIMO system as shown in Figure 1, where M APs equipped with single-antenna are randomly distributed and serve K single-antenna terminal users. The uplink and downlink data transmission of the system adopts the time division duplex (TDD) mode so that all APs can provide data services for users at the same time. Each AP is connected to the CPU through a backhaul network that offers infinite capacity.

The channel coefficient between the k-th user and the m-th AP is denoted as $g_{mk}$, which is modeled as

(1) $$g_{mk}=\beta_{mk}^{1/2}h_{mk}$$

where $\beta_{mk}$ represents the large-scale fading coefficient and $h_{mk}$ represents the small-scale fading coefficient that follows independent and identically distributed (i.i.d.) $CN\left(0,1\right)$.

### 4.1. Uplink Pilot Training Phase and Channel Estimation

In the coherence interval $\tau_{c}$, the pilot symbols selected from a matrix $\Phi \in {\mathbb{C}}^{\tau_{p}\times \tau_{p}}$ are used to estimate the channel coefficients; the pilot of the k-th user is expressed as $\varphi_{k}\in {\mathbb{C}}^{1\times \tau_{p}}$, where $||\varphi_{k}^{H}\varphi_{k^{\prime }}|{|}^{2}=1$ if $\varphi_{k}=\varphi_{k^{\prime }}$, otherwise, $||\varphi_{k}^{H}\varphi_{k^{\prime }}|{|}^{2}=0$. In the uplink training phase, the m-th AP receives

(2) $$y_{p,m}=\sqrt{\tau_{p}\rho_{p}}\sum_{k=1}^{K}g_{mk}\varphi_{k}+n_{p,m}$$

where $\rho_{p}$ represents the normalized signal-to-noise ratio (SNR) of each pilot symbol, $\tau_{p}$ is the upper bounded number of orthogonal pilots, and $n_{p,m}$ is an additive noise vector with i.i.d $CN\left(0,1\right)$ elements.

Referring to [11], the channel coefficient between the k-th user and the m-th AP is estimated through the minimum mean square error (MMSE) estimator, which is given as

(3) $${\hat{g}}_{mk}=c_{mk}(\sqrt{\tau_{p}\rho_{p}}g_{mk}+\sqrt{\tau_{p}\rho_{p}}\sum_{k\prime \neq k}^{K}g_{mk\prime }\varphi_{k}^{H}\varphi_{k\prime }+\varphi_{k}^{H}n_{p,m})$$

where $c_{mk}≜\frac{\sqrt{\tau_{p}\rho_{p}}\beta_{mk}}{\tau_{p}\rho_{p}\sum_{k\prime =1}^{K}\beta_{mk\prime }{\left|\varphi_{k}^{H}\varphi_{k\prime }\right|}^{2}+1}$.

The mean square of the estimated channel vector ${\hat{g}}_{mk}$ is

(4) $$\begin{array}{ll} \gamma_{mk} & ≜E\left\{{\left|{\hat{g}}_{mk}\right|}^{2}\right\}\\ & =\frac{\tau_{p}\rho_{p}\beta_{mk}^{2}}{\tau_{p}\rho_{p}\sum_{k\prime =1}^{K}\beta_{mk\prime }{\left|\varphi_{k\prime }^{H}\varphi_{k}\right|}^{2}+1}\\ & =\sqrt{\tau_{p}\rho_{p}}\beta_{mk}c_{mk} \end{array}$$

Let ${\tilde{g}}_{mk}=g_{mk}-{\hat{g}}_{mk}$ represent the error of channel estimation, which is independent of ${\hat{g}}_{mk}$ and ${\tilde{g}}_{mk}∼CN\left(0,\beta_{mk}-\gamma_{mk}\right)$. From Equation (4), it can be seen that the main reason for causing channel estimation errors, namely pilot contamination, is this item $\sum_{k\prime =1}^{K}\beta_{mk\prime }{\left|\varphi_{k\prime }^{H}\varphi_{k}\right|}^{2}$. Therefore, to reduce pilot contamination, it is necessary to minimize this item and increase system network throughput and user spectrum efficiency.

### 4.2. Uplink Data Transmission Phase

In the uplink data transmission phase of the cell-free massive MIMO system, all users simultaneously send their data to all APs. The received signal at the m-th AP is given by

(5) $$y_{u,m}=\sqrt{\rho_{u}}\sum_{k=1}^{K}g_{mk}\sqrt{\eta_{k}^{u}}q_{k}+n_{u,m}$$

where $q_{k}$ is the symbol sent by the k-th user, satisfies $E\left\{{\left|q_{k}\right|}^{2}\right\}=1$, $\eta_{k}^{u}$ is the data power control coefficient, satisfies $0\leq \eta_{k}^{u}\leq 1$, $\rho_{u}$ is the normalized uplink SNR, and $n_{u,m}∼CN\left(0,1\right)$ is additive noise.

To detect the symbol sent by the k-th user, the m-th AP multiplies the received signal $y_{u,m}$ with the conjugate of its channel estimate ${\hat{g}}_{mk}$ to the CPU using a maximum ratio (MR) combiner through the backhaul network.

The combined signal $r_{k}^{u}=\sum_{m=1}^{M}{\hat{g}}_{mk}^{*}y_{u,m}$ of the k-th user received at the CPU, using the analysis method of [35], is expressed as

(6) $$\begin{array}{ll} r_{k}^{u} & =\sum_{m=1}^{M}{\hat{g}}_{mk}y_{u,m}\\ & =\sum_{k\prime =1}^{K}\sum_{m=1}^{M}\sqrt{\rho_{u}\eta_{k\prime }^{u}}{\hat{g}}_{mk}^{*}g_{mk\prime }q_{k\prime }+\sum_{m=1}^{M}{\hat{g}}_{mk}^{*}w_{u,m}\\ & =\underset{{\mathrm{DS}}_{k}}{\underset{︸}{\sqrt{\rho_{u}\eta_{k}^{u}}q_{k}E\left\{\sum_{m=1}^{M}g_{mk}{\hat{g}}_{mk}^{*}\right\}}}\\ & +\underset{{\mathrm{BU}}_{k}}{\underset{︸}{\sqrt{\rho_{u}\eta_{k}^{u}}q_{k}\left(\sum_{m=1}^{M}g_{mk}{\hat{g}}_{mk}^{*}-E\left\{\sum_{m=1}^{M}g_{mk}{\hat{g}}_{mk}^{*}\right\}\right)}}\\ & +\underset{{\mathrm{MUI}}_{k}}{\underset{︸}{\sqrt{\rho_{u}}\sum_{m=1}^{M}\sum_{k^{\prime }\neq k}^{K}\sqrt{\eta_{k\prime }^{u}}g_{mk}{\hat{g}}_{mk^{\prime }}^{*}q_{k\prime }}}+{\hat{g}}_{mk}^{*}n_{u,k} \end{array}$$

where ${\mathrm{DS}}_{k}$ and ${\mathrm{BU}}_{k}$ represent the desired signal (DS) and beamforming uncertainty (BU) for the k-th user, respectively, and ${\mathrm{MUI}}_{k}$ denotes the multiuser interference (MUI) caused by the other pilot-reuse users.

The beamforming uncertainty (BU) component, multiuser interference (MUI) component, and noise are considered effective noise, so the corresponding achievable uplink rate of the k-th user can be formulated as

(7) $$R_{k}^{u}=\frac{1-\tau_{p}/\tau_{c}}{2}\log_{2}\left(1+\frac{\rho_{u}\eta_{k}^{u}{\left(\sum_{m=1}^{M}\gamma_{mk}\right)}^{2}}{\rho_{u}\sum_{k^{\prime }\neq k}^{K}\eta_{k\prime }^{u}{\left(\sum_{m=1}^{M}\gamma_{mk}\frac{\beta_{mk\prime }}{\beta_{mk}}\right)}^{2}{\left|\varphi_{k}^{H}\varphi_{k\prime }\right|}^{2}+\rho_{u}\sum_{k\prime =1}^{K}\eta_{k\prime }^{u}\sum_{m=1}^{M}\gamma_{mk}\beta_{mk\prime }+\sum_{m=1}^{M}\gamma_{mk}}\right)$$

### 4.3. Downlink Data Transmission Phase

The APs receive downlink data signals encoded by the CPU and then perform transmit precoding based on local channel information, using conjugate beamforming to transmit signals to all users.

The transmitted signal from the m-th AP is given by

(8) $$x_{m}=\sqrt{\rho_{d}}\sum_{k=1}^{K}\sqrt{\eta_{mk}^{d}}{\hat{g}}_{mk}^{*}q_{k}$$

where $q_{k}$, which satisfies $E\left\{{\left|q_{k}\right|}^{2}\right\}=1$, k=1,…,K is the symbol sent to the k-th user, $\eta_{mk}^{d}$, m=1,2,…,M, k=1,…,K is the power control coefficient, and $\rho_{d}$ is the normalized downlink SNR at each AP.

The received signal at the k-th user is given by

(9) $$\begin{array}{l} r_{k}^{d}=\sum_{m=1}^{M}g_{mk}x_{m}+n_{d,k}\\ \;\;\;=\sqrt{\rho_{d}}\sum_{m=1}^{M}\sum_{k\prime =1}^{K}\sqrt{\eta_{mk\prime }^{d}}g_{mk}{\hat{g}}_{mk\prime }^{*}q_{k\prime }+n_{d,k} \end{array}$$

where $w_{d,k}$ is additive $CN\left(0,1\right)$ noise at the k-th user.

The corresponding achievable downlink rate of the k-th user can be formulated as

(10) $$R_{k}^{d}=\frac{1-\tau_{p}/\tau_{c}}{2}\log_{2}\left(1+\frac{\rho_{d}{\left(\sum_{m=1}^{M}\sqrt{\eta_{mk}^{d}}\gamma_{mk}\right)}^{2}}{\rho_{d}\sum_{k^{\prime }\neq k}^{K}{\left(\sum_{m=1}^{M}\sqrt{\eta_{mk^{\prime }}^{d}}\gamma_{mk^{\prime }}\frac{\beta_{mk}}{\beta_{mk^{\prime }}}\right)}^{2}{\left|\varphi_{k}^{H}\varphi_{k\prime }\right|}^{2}+\rho_{d}\sum_{k\prime =1}^{K}\sum_{m=1}^{M}\eta_{mk\prime }^{d}\gamma_{mk\prime }\beta_{mk}+1}\right)$$

### 4.4. Large-M Analysis

In this section, we analyze the performance of a cell-free massive MIMO system when the number of APs is very large.

In the downlink, using the analysis method of [35], the k-th user’s received signal $r_{k}^{d}$ can be represented as

(11) $$\begin{array}{l} r_{k}^{d}=\underset{{\mathrm{DS}}_{k}}{\underset{︸}{\sqrt{\rho_{d}}\sum_{m=1}^{M}\sqrt{\eta_{mk}^{d}}g_{mk}{\hat{g}}_{mk}^{*}q_{k}}}\\ \;+\underset{{\mathrm{MUI}}_{k}}{\underset{︸}{\sqrt{\rho_{d}}\sum_{m=1k}^{M}\sum_{k\prime \neq k}^{K}\sqrt{\eta_{mk\prime }^{d}}g_{mk}{\hat{g}}_{mk\prime }^{*}q_{k\prime }}}+n_{d,k} \end{array}$$

By using the channel estimates in Equation (3), we have

(12) $$\begin{array}{l} \sum_{m=1}^{M}\sqrt{\eta_{mk\prime }^{d}}g_{mk}{\hat{g}}_{mk\prime }^{*}\\ ={\sum_{m=1}^{M}\sqrt{\eta_{mk\prime }^{d}}c_{mk\prime }g_{mk}\left(\sqrt{\tau_{p}\rho_{p}}\sum_{k\prime \prime =1}^{K}g_{mk\prime \prime }\varphi_{k\prime }^{H}\varphi_{k\prime \prime }+{\tilde{n}}_{p,mk\prime }\right)}^{*}\\ =\sqrt{\tau_{p}\rho_{p}}\sum_{m=1}^{M}\sqrt{\eta_{mk\prime }^{d}}c_{mk\prime }{\left|g_{mk}\right|}^{2}\varphi_{k\prime }^{T}\varphi_{k}^{*}\\ \;+\sqrt{\tau_{p}\rho_{p}}\sum_{k\prime \prime \neq k}^{K}\sum_{m=1}^{M}\sqrt{\eta_{mk\prime }^{d}}c_{mk\prime }g_{mk}g_{mk\prime \prime }^{*}\varphi_{k\prime }^{T}\varphi_{k\prime \prime }^{*}\\ \;+\sum_{m=1}^{M}\sqrt{\eta_{mk\prime }^{d}}c_{mk\prime }g_{mk}{\tilde{n}}_{p,mk\prime }^{*} \end{array}$$

where ${\tilde{w}}_{p,mk\prime }^{*}≜\varphi_{k\prime }^{H}w_{p,m}$. By Tchebyshev’s theorem [36], we have

(13) $$\begin{array}{l} \frac{1}{M}\sum_{m=1}^{M}\sqrt{\eta_{mk\prime }^{d}}g_{mk}{\hat{g}}_{mk\prime }^{*}-\frac{1}{M}\sqrt{\tau_{p}\rho_{p}}\sum_{m=1}^{M}\sqrt{\eta_{mk\prime }^{d}}c_{mk\prime }\beta_{mk}\varphi_{k\prime }^{T}\varphi_{k}^{*}\overset{P}{\underset{M\to \infty }{\to }}0 \end{array}$$

Using (13), we can obtain the following results

(14) $$\begin{array}{l} \frac{1}{M}DS_{k}-\frac{1}{M}\sqrt{\tau_{p}}\rho_{p}\sum_{m=1}^{M}\sqrt{\eta_{mk\prime }^{d}}c_{mk}\beta_{mk}q_{k}\overset{P}{\underset{M\to \infty }{\to }}0\\ \frac{1}{M}MUI_{k}-\frac{1}{M}\sqrt{\tau_{p}}\rho_{p}\sum_{m=1}^{M}\sum_{k\prime \neq k}^{K}\sqrt{\eta_{mk\prime }^{d}}c_{mk\prime }\beta_{mk}\varphi_{k\prime }^{T}\varphi_{k}^{*}q_{k\prime }\overset{P}{\underset{M\to \infty }{\to }}0 \end{array}$$

The above formula shows that when M→∞ the received signal only includes the desired signal and interference originating from the non-orthogonality of the pilot sequence:

(15) $$\frac{r_{k}^{d}}{M}-\frac{\sqrt{\tau_{p}}\rho_{p}}{M}\left(\sum_{m=1}^{M}\sqrt{\eta_{mk}^{d}}c_{mk}\beta_{mk}q_{k}\right.\left.+\sum_{m=1}^{M}\sum_{k\prime \neq k}^{K}\sqrt{\eta_{mk\prime }^{d}}c_{mk\prime }\beta_{mk}\varphi_{k\prime }^{T}\varphi_{k}^{*}q_{k\prime }\right)\overset{P}{\underset{M\to \infty }{\to }}0$$

If the pilot symbols are pairwisely orthogonal, i.e., $\varphi_{k\prime }^{H}\varphi_{k}=0\;\mathrm{for}\;k\neq k\prime$, then the received signal becomes free of interference and noise:

(16) $$\frac{r_{k}^{d}}{M}-\frac{\sqrt{\tau_{p}}\rho_{p}}{M}\left(\sum_{m=1}^{M}\sqrt{\eta_{mk}^{d}}c_{mk}\beta_{mk}q_{k}\right)\overset{P}{\underset{M\to \infty }{\to }}0$$

The analysis of the uplink is similar. From the above analysis, it can be concluded that if M→∞ the channel between the user and the AP will be orthogonal. Therefore, noncoherent interference, small-scale fading, and noise disappear through matched filtering using conjugate beamforming, respectively. The remaining issue is pilot contamination, which refers to the interference that arises when multiple users reuse pilots during the uplink pilot training phase.

## 5. Joint User Clustering and Weighted Graph Coloring Based Pilot Assignment

### 5.1. User Clustering Based on AP Selection

If the number of pilots is far less than the number of users, a full service-based AP selection scheme can lead to serious pilot contamination issues. Therefore, before assigning pilots to users, suitable algorithms should be chosen to assign a specific set of APs to each user. Based on this, we separate all users into different clusters, so users connected to the same APs are grouped into the same cluster.

We select an exclusive AP and shared APs for each user based on their large-scale fading coefficient. The exclusive APs ensure the service quality of the APs that connect to users with the maximum large-scale fading coefficient. As observed in [28], if a user connects to only one AP, their throughput will greatly decrease. Hence, shared APs enable the system to perform better.

Each user selects an AP with the largest large-scale fading coefficient from the set of available service Aps’ $M_{AP}$ to be their exclusive AP. Only one user is allowed to be served by each exclusive AP. For each user, select shared APs with high large-scale fading coefficient values between that user and each respective AP. The method for selecting shared APs is as follows:

(17) $$\sum_{m\in S_{s}^{k}}^{M}{\tilde{\beta }}_{mk}\geq \alpha \sum_{m\prime \in M_{AP}}^{M}\beta_{m\prime k}$$

where $S_{s}^{k}$ represents the k-th user’s shared AP set, ${\tilde{\beta }}_{mk}\in \left\{{\tilde{\beta }}_{1k},{\tilde{\beta }}_{2k},\ldots ,{\tilde{\beta }}_{Mk}\right\}$ is the ranking result of the k-th user and all APs in descending order of the large-scale fading coefficient $\beta_{mk}$, and α represents the threshold adjustment factor for the k-th user’s selection of shared APs.

Based on the above analysis, the AP set for the k-th user can be expressed as follows:

(18) $$a_{mk}=\left\{\begin{array}{c} 1,\;\mathrm{if}\;m\in S_{e}^{k}\;\mathrm{or}\;m\in S_{s}^{k}\\ 0,\;\;\;\mathrm{otherwise}\; \end{array}\right.$$

where if $a_{mk}=1$ it indicates that the k-th user selected the m-th AP as its exclusive AP or shared AP, otherwise it indicates that the k-th user did not select the m-th AP.

To sum up, the k-th user’s AP set $S^{k}=S_{e}^{k}+S_{s}^{k}$ and the AP service matrix $A_{M\times K}$ can be obtained by using Equations (17) and (19). The matrix can be expressed as follows:

(19) $$A_{M\times K}=\left(\begin{array}{ccc} a_{11} & \ldots & a_{1K}\\ \vdots & \ddots & \vdots \\ a_{M1} & \cdots & a_{MK} \end{array}\right)$$

The corresponding achievable uplink and downlink rates for the k-th user can be re-derived based on the aforementioned analysis, as shown in (20) and (21), respectively.

(20) $$R_{k}^{u}=\frac{1-\tau_{p}/\tau_{c}}{2}\log_{2}\left(1+\frac{\rho_{u}\eta_{k}^{u}{\left(\sum_{m\in S^{k}}^{M}\gamma_{mk}\right)}^{2}}{\sum_{k^{\prime }=1}^{K}\rho_{u}\eta_{k^{\prime }}^{u}{\left(\sum_{m\in S^{k}}\gamma_{mk^{\prime }}\frac{\beta_{mk\prime }}{\beta_{mk}}\right)}^{2}{\left|\varphi_{k}^{H}\varphi_{k\prime }\right|}^{2}+\sum_{k\prime =1}^{K}\rho_{u}\eta_{k\prime }^{u}\sum_{m\in S^{k}}\gamma_{mk\prime }\beta_{mk}+\sum_{m\in S^{k}}\gamma_{mk}}\right)$$

(21) $$R_{k}^{d}=\frac{1-\tau_{p}/\tau_{c}}{2}\log_{2}\left(1+\frac{\rho_{d}{\left(\sum_{m\in S^{k}}^{M}\sqrt{\eta_{mk}^{d}}\gamma_{mk}\right)}^{2}}{\sum_{k^{\prime }=1}^{K}\rho_{d}{\left(\sum_{m\in S^{k}}^{M}\sqrt{\eta_{mk^{\prime }}^{d}}\gamma_{mk^{\prime }}\frac{\beta_{mk}}{\beta_{mk^{\prime }}}\right)}^{2}{\left|\varphi_{k}^{H}\varphi_{k\prime }\right|}^{2}+\sum_{k\prime =1}^{K}\rho_{d}\sum_{m\in S^{k}}^{M}\eta_{mk\prime }^{d}\gamma_{mk\prime }\beta_{mk}+\sigma_{d,k}^{2}}\right)$$

Equations (20) and (21) show that pilot contamination occurs when multiple users connected to the same APs reuse the same pilot. Therefore, to cluster users who are connected to the same APs as much as possible into the same user cluster, we further introduce the K-means clustering algorithm, which uses the user’s AP selection in addition to their geographic location information.

To improve the gain of pilot reuse, the number of users divided into each cluster is at most equal to the number of available orthogonal pilots. So, all users can be divided into $\tilde{K}$ clusters, $\tilde{K}=\left\lceil K/\tau_{p}\right\rceil$. Then, a pilot assignment algorithm can be utilized to assign orthogonal pilots to users of each cluster to effectively alleviate pilot contamination.

To avoid inaccurate allocation results caused by the random selection of initial centroids, the proposed algorithm selects users whose AP set does not intersect with the AP sets of other users as the initial centroids. The remaining users associate themselves with the centroid closest to their geographical location.

The process of user clustering algorithm based on AP selection is shown in Algorithm 1.
**Algorithm 1:** User Clustering Algorithm Based on AP Selection**Input:** M, K, large scale fading coefficient matrix $B_{M\times K}$, select threshold α**Output: User clustering results**$C_{\tilde{K}\times \tau_{p}}$1. Initialize: $\;\;S^{k}=0$, step=0, $\tilde{K}=K/\tau_{p}$, num=1
2. **for** k=1:K **do**3.  Sort the k-th user’s large-scale fading coefficient in descending order $\left[B_{M\times K}(:,k),index\right]=sort(B_{M\times K}(:,k),\prime descend\prime )$.4.  Add the AP corresponding to the largest large-scale fading coefficient to the user’s exclusive AP set $S_{e}^{k}\leftarrow B_{M\times K}\left(index(1),k\right)$$B_{M\times K}\left(index(1),\sim \right)=0$.5. **end for**6. **for** k=1:K **do**7. Select a shared AP set $S_{s}^{k}$ for the k-th user according to Equation (17). 8.  Obtain the AP set $S^{k}$ of the k-th user based on the shared AP set $S_{s}^{k}$ and the exclusive AP set $S_{e}^{k}$ of the k-th user.9. **end for**10. **while** $num<\tilde{K}$ **do**11.   Select the k-th user that is different from the end of the previous cycle to join the centroid set randomly Q←k.12.   **for** i=1:K **do**13.   **if** $S^{i}\cap S^{k}=\emptyset$ **then**14.    Add the i-th user to the centroid set Q←i,num=num+1.15.   **end if**16.   **end for**17. **end while**18. **for** i=1:K **do**19.  Calculate the Euclidean distance between the k-th user and each centroid and assign the k-th user to the cluster $C_{i}$ where the nearest centroid is located. If the number of users in this cluster has exceeded the number of available orthogonal pilots, remove this centroid and select the next centroid.20. **end for**21. Update the centroid based on the result of the current iteration $\;\;\;\;\;\;\;\;x_{Q_{i}^{\prime }}=\frac{1}{\left|C_{i}\right|}\sum_{k\in C_{i}}x_{k},y_{Q_{i}^{\prime }}=\frac{1}{\left|C_{i}\right|}\sum_{k\in C_{i}}y_{k}\;,\forall i=1,2,\ldots ,\tilde{K}$.22. Increment the number of iterations by one step=step+1.23. If the centroids no longer change or the number of iterations is reached, the algorithm ends, otherwise it goes to 18.

### 5.2. Joint User Clustering and Graph Coloring Based Pilot Assignment

After completing user clustering, orthogonal pilots can be assigned to users in the same cluster to reduce intra-cluster interference. However, since all users are usually not connected to the same APs and are not within the same cluster, both intra-cluster and inter-cluster interference must be considered during the pilot allocation. We first consider the interference among inter-cluster users by mapping it to a graph coloring problem and constructing a weighted pilot contamination graph for inter-cluster users. Pilots are then assigned to inter-cluster users to maximize the uplink convergence throughput rate of inter-cluster users and, finally, orthogonal pilots are assigned to users within each cluster, thereby improving the pilot contamination of the entire network.

#### 5.2.1. Weighted Pilot Contamination Graph

In this paper, we introduce the terms “inter-cluster users” (to refer to users who are connected to the same APs but not in the same cluster) and “intra-cluster users” (to refer to users who are connected to the same APs and in the same cluster), which can be given as

(22) $$\left\{\begin{array}{l} S^{k}\cap S^{C_{k^{\prime }}}\neq \emptyset :\mathrm{inter}-\mathrm{cluster}\;\mathrm{users}\\ S^{k}\cap S^{C_{k^{\prime }}}=\emptyset :\mathrm{intra}-\mathrm{cluster}\;\mathrm{users} \end{array}\right.$$

where $S^{C_{k\prime }}$ represents the AP set for any user in a different cluster than the k-th user.

**Figure 2:**
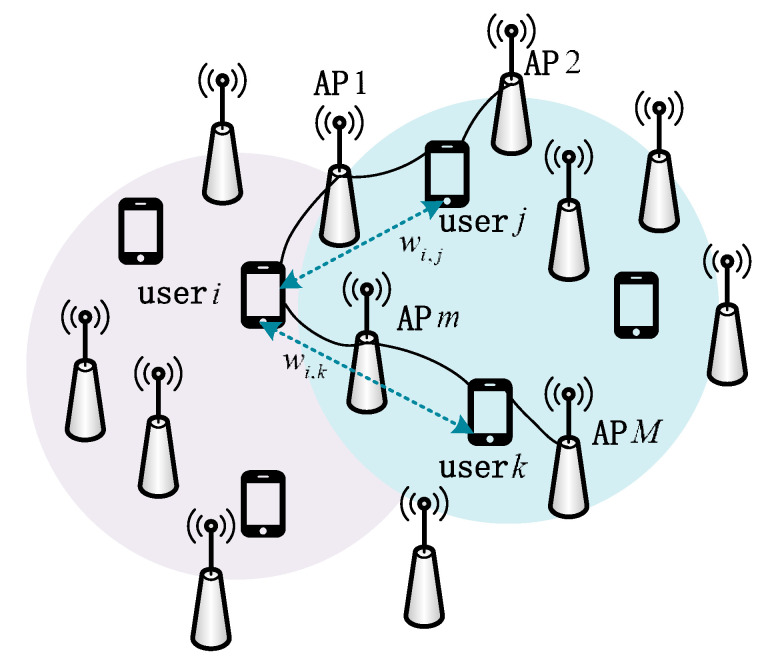
Schematic diagram of inter-cluster users’ interference.

Figure 2 shows that users located within the same color circle represent intra-cluster users. The figure also illustrates that there is an intersection of AP sets between the two clusters. Specifically, the i-th user from one cluster and the j-th user, the k-th user from the other cluster are connected to the same APs. If the i-th user and one of the users from the other cluster share the same pilot, it leads to pilot contamination inevitably. Previous studies, such as [28], did not address pilot contamination separately but instead considered pilot assignment for the entire system, leading to high complexity and negligible improvements in performance. To address this issue, we propose an approach that uses a graph coloring algorithm to assign pilots to inter-cluster users and assigns orthogonal pilots to the remaining intra-cluster users in each cluster.

This section addresses the pilot assignment problem for inter-cluster users by converting it into an optimization problem that maximizes the achievable uplink rate:

(23) $$P1:\underset{p}{\max}f\left(p\right)=\sum_{k\in U_{e}}R_{k}^{u}$$

where $U_{e}$ is the set of inter-clusters including all inter-cluster users and $p=\left\{\varphi_{k},k\in \left\{1,2,\ldots K\right\}\right\}$ represents the pilot assignment strategy.

Once APs are selected, we can obtain a valid set of service APs $S_{k}=\left\{k^{\left(1\right)},k^{\left(2\right)},\ldots k^{\left(M_{k}\right)}\right\}$ for the k-th user. In contrast to [28], we introduce weights between inter-cluster users based on AP selection using large-scale fading coefficients. If the distance between the interfering $k^{\prime }-\mathrm{th}$ user and the m-th AP, $m\in S_{k}$, is shorter than the distance between the k-th user and the m-th AP, the $k^{\prime }-\mathrm{th}$ user will pose serious potential interference to the k-th user. For the service AP and the target k-th user, the gain obtained is proportional to ${\left|\beta_{mk}\right|}^{2}$ and the interference is proportional to ${\left|\beta_{mk^{\prime }}\right|}^{2}$. The ratio ${\left|\beta_{mk^{\prime }}/\beta_{mk}\right|}^{2}$ quantifies the interference from the $k^{\prime }-\mathrm{th}$ user. Consequently, the interference graph considers the weight between two users mainly based on large-scale fading coefficients. Taking the k-th user and the $k^{\prime }-\mathrm{th}$ user as an example, their interference metric, $w_{k,k^{\prime }}$, can be expressed as

(24) $$w_{k,k^{\prime }}={\left|\sum_{m\in \left(S_{k}\cap S_{k^{\prime }}\right)}\frac{\beta_{mk^{\prime }}}{\sum_{m\in S_{k}}\beta_{mk}}\right|}^{2}+{\left|\sum_{m^{\prime }\in \left(S_{k}\cap S_{k^{\prime }}\right)}\frac{\beta_{mk}}{\sum_{m^{\prime }\in S_{k^{\prime }}}\beta_{m^{\prime }k^{\prime }}}\right|}^{2}$$

where $w_{k,k^{\prime }}$ includes two parts: the interference caused by the $k^{\prime }-\mathrm{th}$ user to the k-th user and the interference caused by the k-th user to the $k^{\prime }-\mathrm{th}$ user.

The larger the value of $w_{k,k^{\prime }}$, the greater the interference between the k-th user and the $k^{\prime }-\mathrm{th}$ user. The weight matrix of the inter-cluster can be expressed as

(25) $$W_{k,k^{\prime }}=\left(\begin{array}{ccc} w_{1,1} & \ldots & w_{1,K_{1}}\\ \vdots & \ddots & \vdots \\ w_{K_{1},1} & \cdots & w_{K_{1},K_{1}} \end{array}\right)$$

where $K_{1}$ is the total number of inter-cluster users.

#### 5.2.2. Pilot Assignment Scheme

For the cell-free massive MIMO system, pilot assignment to the vertices begins after constructing the interference graph. A greedy approach is employed to allocate pilots to users based on the magnitude of the interference value, in descending order. The proposed GC-UC pilot assignment algorithm is described and analyzed in Algorithm 2.

Step 1 involves calculating the inter-cluster and intra-cluster users based on system parameters and initializing the pilot assignment matrix.

Steps 2 to 8 include calculating the interference weight between two inter-cluster users, computing the total interference value for each user, and ranking the results in descending order.

Steps 9 to 11 involve initially assigning orthogonal pilots to users whose sum of interference ranks in the top $\tau_{p}$.

Steps 12 to 18 involve calculating the preference of remaining inter-cluster users for each pilot. The preference is calculated by summing the interference weight between the target user and the user using the current pilot. The smaller the sum of the interference weight, the higher the preference, and vice versa. The target user is assigned the pilot with the highest preference.

Next, pilots are assigned to intra-cluster users. Because the number of users in each cluster is not greater than the number of orthogonal pilots available, intra-cluster users can be assigned with orthogonal pilots. Steps 19 to 24 entail gathering statistics on the pilots that were not assigned to inter-cluster users in each cluster and sequentially assigning them to intra-cluster users.
**Algorithm 2:** Joint User Clustering and Graph Coloring based Pilot Assignment, UC-GCInput: $M,K,S,C,\tau_{p},\beta_{mk}$
Output: pilot assignment matrix P
1. Initialize:  Inter-cluster users $U_{ed}=\left\{U_{k}:1\leq k\leq K_{2}\right\}$
  the number of the inter-cluster users $K_{1},K_{1}+K_{2}=K$
  User set of subgraphs $V_{i}=\emptyset ,\mathrm{for}\;1\leq i\leq \tau_{p}$
2. **for** $i=1:K_{1}$ **do**3.  **for** $j=2:K_{1}$ **do**4.   Calculate the interference value between two users $w_{k,k^{\prime }}={\left|\sum_{m\in \left(S_{k}\cap S_{k^{\prime }}\right)}\frac{\beta_{mk^{\prime }}}{\sum_{m\in S_{k}}\beta_{mk}}\right|}^{2}+{\left|\sum_{m^{\prime }\in \left(S_{k}\cap S_{k^{\prime }}\right)}\frac{\beta_{mk}}{\sum_{m^{\prime }\in S_{k^{\prime }}}\beta_{m^{\prime }k^{\prime }}}\right|}^{2}$.5.  **end for**6.  Calculate the sum of the interference value of the k-th user $W_{\mathrm{sum}}(k)$.7. **end for**8.  Sort $W_{\mathrm{sum}}$ in descending $\left[W_{sum},index]=sort(W_{sum},\prime descending\prime \right)$.9. **for** $j=1:\tau_{p}$ **do**
10.  Assign the j-th pilot to the user $U_{k}$ corresponding to $W_{sum}\left(j\right)$, $V_{i}=V_{i}+U_{k}$, $U_{ed}=U_{ed}-U_{k}$.11. **end for**12. **while** $U_{ed}\neq \emptyset$ **do**13.  Arbitrarily select $U_{k}\in U_{ed}$.14.  **for** $j=1:\tau_{p}$ **do**15.   Calculate $E_{k,i}=\sum_{\left\{U_{k^{\prime }}\right\}\in V_{i}}w_{k,k^{\prime }}$.16.  **end for**17.  $V_{i}=V_{i}+U_{k}$ with $i^{\prime }=\underset{i}{\arg\min}E_{k,i}$, $U_{ed}=U_{ed}-U_{k}$.18. **end while**19. **for** $i=1:K_{2}$ **do**20.  **for** $j=1:\tau_{p}$ **do**21.   Judge whether the j-th pilot is assigned to the user in the same cluster.22.   If not assigned, assign the j-th pilot to the i-th user.23.  **end for**24. **end for**25. Update and return P according to $V_{i},\;\mathrm{for}\;1\leq i\leq \tau_{p}$.

## 6. Result and Discussion

### 6.1. Simulation Setup and Parameters

In this section, we evaluate the performance of the proposed scheme. The APs and users of the cell-free massive MIMO system are randomly located within a square area of $1\times 1{\;\mathrm{km}}^{2}$. The perfect backhaul link to the CPU is considered without accounting for power consumption. The per-user throughput considered as the performance metric is defined as $T_{k}^{u(d)}=B\cdot R_{k}^{u(d)}$, where B represents the spectral bandwidth.

The coefficient models the path loss and shadow fading, according to

(26) $$\beta_{mk}={\mathrm{PL}}_{mk}\cdot 10^{\frac{\sigma_{sh}z_{mk}}{10}}$$

where ${\mathrm{PL}}_{mk}$ denotes path loss from the k-th user to the m-th AP, $10^{\frac{\sigma_{sh}z_{mk}}{10}}$ denotes shadow fading with standard deviation $\sigma_{sh}=8\;\mathrm{dB}$, and $z_{mk}∼N\left(0,1\right)$.

For the path loss, we use the following path loss mode [37]:

(27) $${\mathrm{PL}}_{\mathrm{mk}}=\left\{\begin{array}{c} -L-15\log_{10}(d_{mk}),\;\;\;\;\;\;if\;d_{mk}>d_{1}\\ -L-15\log_{10}(d_{1})-20\log_{10}(d_{mk}^{2}),\;if\;d_{0}<d_{mk}\leq d_{1}\\ -L-15\log_{10}(d_{1})-20\log_{10}(d_{0}),\;\;\;if\;d_{mk}\leq d_{0} \end{array}\right.$$

where $d_{mk}$ denotes the distance between the k-th user and the m-th AP. The quantity of L is

(28) $$\begin{array}{l} L≜4.63+33.9\log_{10}(f)-13.82\log_{10}(H_{b})\\ \;-(1.1\log_{10}(f)-0.7)H_{m}+(1.56\log_{10}(f)-0.8) \end{array}$$

where f is the carrier frequency and $H_{m}$, $H_{b}$ denotes the user and AP antenna heights, respectively.

The main simulation parameters are listed in Table 1.

**Figure 3:**
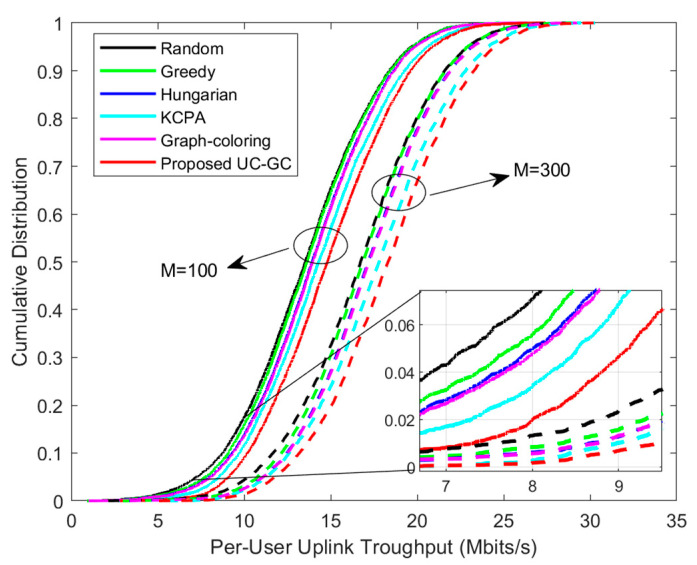
Cumulative distribution of per-user uplink throughput for different pilot assignment schemes with $K=40,\;\tau_{p}=10$.

**Figure 4:**
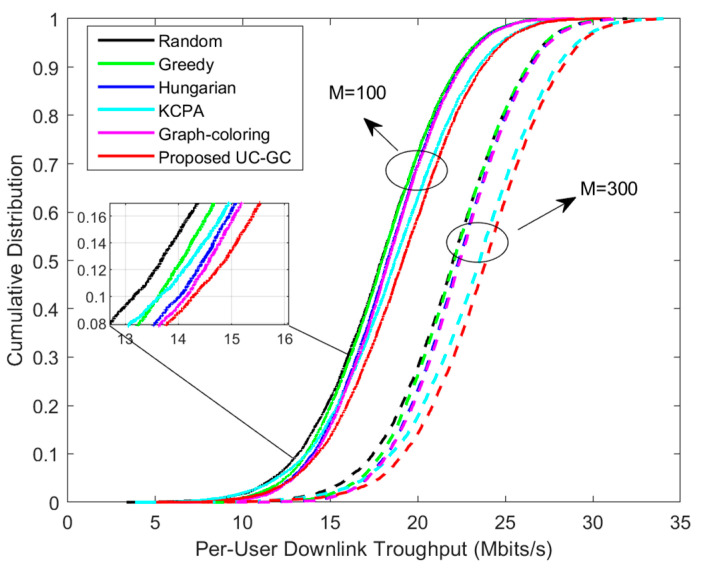
Cumulative distribution of per-user downlink throughput for different pilot assignment schemes with $K=40,\;\tau_{p}=10$.

**Figure 5:**
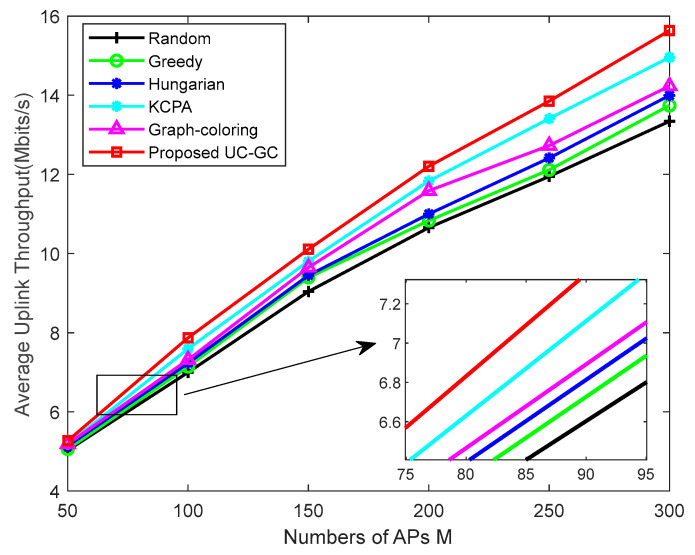
Average uplink throughput against the number of APs M with $K=40,\;\tau_{p}=10$.

**Figure 6:**
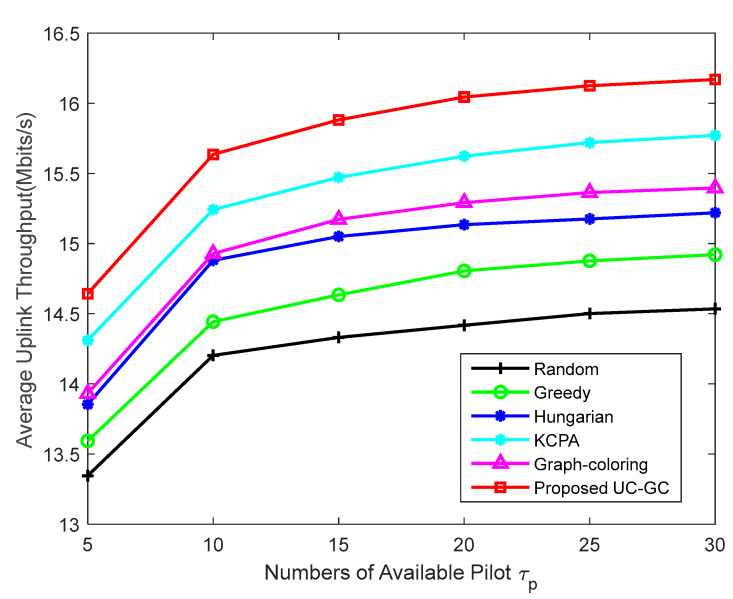
Average uplink throughput against the number of pilots $\tau_{p}$ with M=300, K=40.

The proposed scheme is compared with five schemes:

(1) Random pilot assignment scheme [11]: Each user connects to all APs and is assigned a pilot randomly. This scheme is marked as “Random” in the figures.
(2) The greedy based pilot assignment scheme [11]: Each user connects to all APs and the data rate for the worst user is iteratively refined using the greedy algorithm idea. This scheme is marked as “Greedy” in the figures.
(3) The Hungarian based pilot assignment scheme [26]: Each user connects to all APs and each user’s pilot is updated iteratively using the Hungarian algorithm idea. This scheme is marked as “Hungarian” in the figures.
(4) The K-means clustering based pilot assignment scheme: The AP selection algorithm proposed in this paper is used to select a set of APs for each user. Then, the users are clustered using the pilot allocation algorithm proposed in [34] and orthogonal pilots are assigned to the users within each cluster with inter-cluster reused pilots. This scheme is marked as “KCPA” in the figures.
(5) The graph coloring based pilot assignment scheme [28]: This scheme utilizes large-scale fading coefficients between the APs and the users to operate an AP selection algorithm. With the resulting AP selection, an interference graph is constructed, which is then utilized to assign pilots to the users. This scheme is marked as “Graph-coloring” in the figures.
(6) The joint user clustering and graph coloring based pilot assignment scheme: The scheme that is proposed in this paper. This scheme is marked as “Proposed UC-GC” in the figures.

### 6.2. Numerical Results and Discussion

Figure 3 and Figure 4 demonstrate the cumulative distribution of per-user uplink throughput and downlink throughput for different pilot assignment schemes, respectively. In the simulation, the total number of users is set to 40, with two cases as (1) M=100 and (2) M=300, where the corresponding parameter sets are: K=40, $\tau_{p}=10$. The results show that the performance of the proposed scheme is significantly superior to other schemes and, as the total number of APs increases, the performance gap between the proposed scheme and others increases. It is running as expected, since the proposed scheme gradually alleviates pilot contamination from three aspects: AP selection, user clustering, and pilot assignment. Our proposed scheme first selects APs that contribute significantly to serving the users through the AP selection algorithm to ensure high-quality communication. Then, the clustering algorithm divides users into distinct groups to simplify the allocation of pilots. Finally, we incorporate the graph coloring algorithm to assign pilots to users based on their relationship, which improves the overall performance of the system.

Figure 5 shows the average uplink throughput varying with the number of APs’ M, where $K=40,\;\tau_{p}=10$. It can be observed that the average uplink throughput of all pilot assignment schemes increases as M increases, because a large number of APs provide better macro diversity gain and the received signal strength increases due to network densification. In addition, as the number of APs increases, the AP selection algorithm we propose can select APs with greater gains for users, so that the average uplink throughput of the proposed scheme is higher than that of the random scheme, the greedy scheme, the Hungarian scheme, the KCPA scheme, and the graph coloring scheme by about 2.30 Mbits/s, 1.90 Mbits/s, 1.64 Mbits/s, 0.68 Mbits/s, and 1.41 Mbits/s, respectively, and the average uplink throughput of the proposed scheme is always higher than that of other schemes, which verifies the effectiveness and superiority of the UC-GC scheme under large-scale AP layout conditions.

Figure 6 investigates the impact of the number of mutually orthogonal pilots $\tau_{p}$ in the case of M=300, K=40. The figure clearly shows the superiority of the proposed scheme over other schemes. Furthermore, the proposed scheme is capable of maintaining its performance, even in scenarios with limited pilot resources. The smaller the number of pilots $\tau_{p}$, the lower the overall performance of the system. This is because the smaller the length of orthogonal pilots, the less time spent on pilot training at each coherent interval and the greater the resulting channel estimation error, leading to serious pilot contamination. In all schemes, when the number of pilots $\tau_{p}$ increases to a certain extent, the average uplink throughput of users increases slowly. Increasing the length of the pilot symbols will inevitably compress the length of the uplink and downlink data symbols due to the limited coherent time, so the overall throughput does not change significantly.

**Figure 7:**
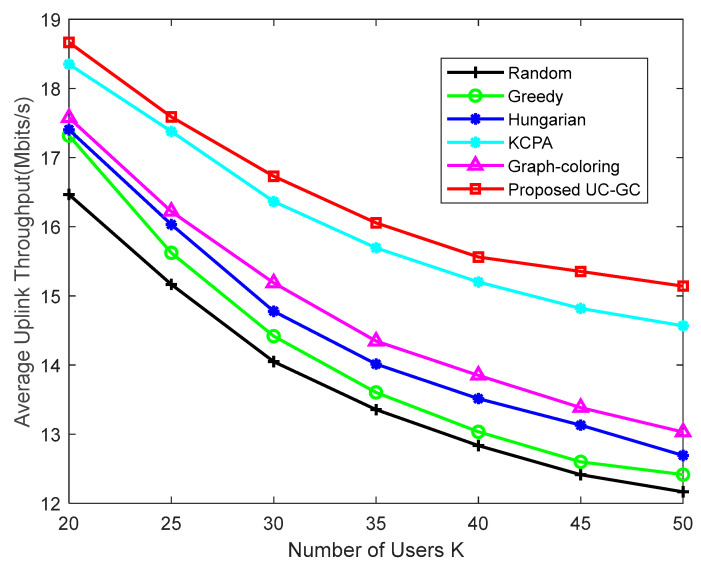
Average uplink throughput against the number of users K with $M=300,\;\tau_{p}=10$.

Figure 7 shows the average uplink throughput of users varying with the number of users K in the case of M=300, $\tau_{p}=10$. As can be seen from the figure, as the number of users increases, pilot reuse becomes more severe, resulting in a decrease in the average uplink throughput of users in all pilot assignment schemes. Existing pilot assignment schemes usually directly assign pilots to all users, which not only increases the complexity of the assignment but also causes more serious interference among users as the number of users increases. The scheme proposed in this paper first performs clustering before pilot assignment, taking into account the situation of each cluster separately and narrowing the range of pilot assignment. We also propose the UC-GC algorithm to assign pilots to different types of users, thereby reducing the complexity of the pilot assignment and effectively alleviating the interference among users and maintaining relatively good performance.

**Figure 8:**
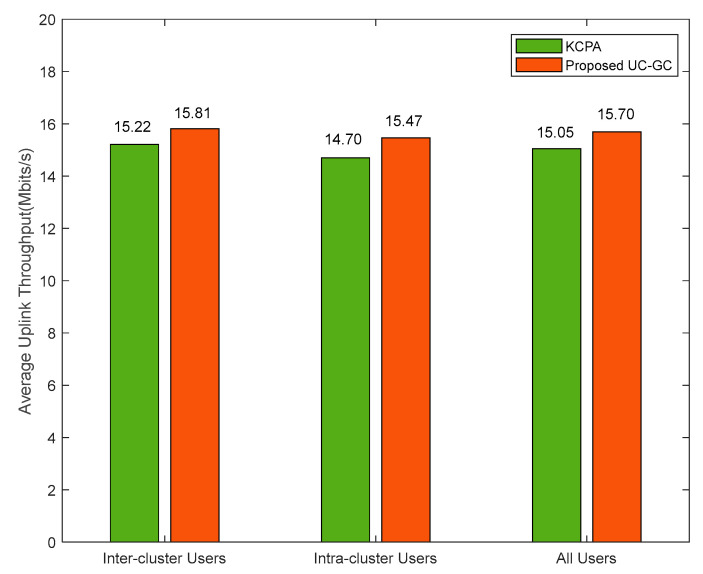
Average uplink throughput of different types of users for different pilot assignment schemes with $M=300,\;K=40,\;\tau_{p}=10$.

Figure 8 shows the comparison of the average uplink throughput of different types of users in the KCPA scheme and the proposed scheme in the case of M=300, K=40, $\tau_{p}=10$. The KCPA algorithm divides users into different clusters and directly assigns pilots to users within each cluster. However, this scheme ignores interference among inter-cluster users, leading to insignificant performance improvement. We propose the UC-GC algorithm that assigns pilots to inter-cluster users with high interference first and then assigns orthogonal pilots to the remaining users based on clustering. The average uplink throughput of the proposed scheme for inter-cluster users, intra-cluster users, and all users is 0.59 Mbit/s, 0.77 Mbit/s, and 0.65 Mbit/s higher than the KCPA scheme, respectively. This indicates that the proposed scheme not only improves the pilot contamination of inter-cluster users but also improves the throughput of intra-cluster users, thereby improving the overall throughput of the entire system.

### 6.3. Complexity Anslysis

The algorithm complexity is evaluated for the particular case with K users, M APs, and τ pilot constraint. The complexity of the greedy scheme is O((2K+1)M). The Hungarian algorithm’s complexity is approximate $O(K\tau^{3})$. The complexity of the KCPA scheme is $O(K^{2}+K\tau )$. If the graph coloring algorithm is directly utilized for pilot assignment for all users, the complexity of the graph coloring scheme is $O(K^{3}\tau )$. In addition, the complexity of the UC-GC scheme is $O(K^{2}+3K+K\tau +2\tau )$. For cell-free massive MIMO systems, M≫K≫τ would be satisfied. Therefore, the proposed scheme has a lower degree of complexity compared with other schemes.

## 7. Conclusions

In this paper, we introduce a novel scheme that blends user clustering and pilot assignment to alleviate the pilot contamination challenge for the cell-free massive MIMO systems. The proposed scheme includes three aspects: AP selection, user clustering, and pilot assignment. First, the improved K-means algorithm is utilized to cluster all users based on the AP selection result, dividing users into intra-cluster and inter-cluster users. Next, the pilot assignment for inter-cluster users is regarded as a graph coloring problem. A weighted pilot contamination graph is structured to describe the dynamic interference relationship among inter-cluster users. Finally, the UC-GC algorithm is proposed to carry out pilot assignments for the cell-free massive MIMO system, which can effectively reduce the search space and the complexity of the pilot assignment. The proposed scheme shows more exceptional performance than the existing methods, particularly when the ratio of the number of APs to the number of users is high.

## Figures and Tables

**Table 1:** Main Simulation System Parameters.

Parameter Name	Symbol	Reference Value
Carrier frequency	f	1.9 GHz
Spectral bandwidth	B	20 MHz
Noise figure (uplink and downlink)	$\sigma_{n}$	9 dB
AP antenna height	$H_{b}$	15 m
User antenna height	$H_{m}$	1.65 m
Coherence time length	$\tau_{c}$	200
Pilot length	$\tau_{p}$	10
Transmit power of pilot symbols and data	$p_{p},p_{d}$	200 mW
Number of APs	M	100, 300
Number of users	K	40

## Data Availability

Not applicable.

## References

[1] Björnson E., Sanguinetti L. (2019). Making cell-free massive MIMO competitive with MMSE processing and centralized implementation. IEEE Trans. Wirel. Commun..

[2] Yu H., Yi X., Caire G. (2022). Topological pilot assignment in large-scale distributed MIMO networks. IEEE Trans. Wirel. Commun..

[3] Wang H., Wang J., Fang J. (2020). Grant-free massive connectivity in massive MIMO systems: Collocated versus cell-free. IEEE Wirel. Commun. Lett..

[4] Ammar H.A., Adve R., Shahbazpanahi S., Boudreau G., Srinivas K.V. (2021). User-centric cell-free massive MIMO networks: A survey of opportunities, challenges and solutions. IEEE Commun. Surv. Tutor..

[5] Ammar H.A., Adve R., Shahbazpanahi S., Boudreau G., Srinivas K.V. (2021). Downlink resource allocation in multiuser cell-free MIMO networks with user-centric clustering. IEEE Trans. Wirel. Commun..

[6] Papazafeiropoulos A., Kourtessis P., Di Renzo M., Chatzinotas S., Senior J.M. (2020). Performance analysis of cell-free massive MIMO systems: A stochastic geometry approach. IEEE Trans. Veh. Technol..

[7] Zhou M., Zhang Y., Cao H., Qiao X., Yang L. Enhanced power allocation algorithms for uplink mixed ADCs massive MIMO systems. Proceedings of the 2019 IEEE Globecom Workshops (GC Wkshps).

[8] Zhang Y., Zhou M., Cao H., Yang L., Zhu H. (2019). On the performance of cell­free massive MIMO with mixed­ADC under Rician fading channels. IEEE Commun. Lett..

[9] Demir Ö.T., Björnson E., Sanguinetti L. (2021). Foundations of user-centric cell-free massive MIMO. Found. Trends Signal Process..

[10] Chen S., Zhang J., Zhang J., Björnson E., Ai B. (2022). A survey on user-centric cell-free massive MIMO systems. Digit. Commun. Netw..

[11] Ngo H.Q., Ashikhmin A., Yang H., Larsson E.G., Marzetta T.L. (2017). Cell-free massive MIMO versus small cells. IEEE Trans. Wirel. Commun..

[12] Nayebi E., Ashikhmin A., Marzetta T.L., Yang H., Rao B.D. (2017). Precoding and power optimization in cell-free massive MIMO systems. IEEE Trans. Wirel. Commun..

[13] Buzzi S., D’Andrea C., Zappone A., D’Elia C. (2019). User-centric 5G cellular networks: Resource allocation and comparison with the cell-free massive MIMO approach. IEEE Trans. Wirel. Commun..

[14] Buzzi S., D’Andrea C. (2017). Cell-free massive MIMO: User-centric approach. IEEE Wirel. Commun. Lett..

[15] Ngo H.Q., Tran L.N., Duong T.Q., Matthaiou M., Larsson E.G. (2017). On the total energy efficiency of cell-free massive MIMO. IEEE Trans. Green Commun. Netw..

[16] Wang R., Shen M., He Y., Liu X. (2021). Performance of cell-free massive MIMO with joint user clustering and access point selection. IEEE Access.

[17] Wei C., Xu K., Xia X., Su Q., Shen M., Xie W., Li C. (2022). User-centric access point selection in cell-free massive MIMO systems: A game-theoretic approach. IEEE Commun. Lett..

[18] Biswas S., Vijayakumar P. (2021). AP selection in Cell-Free Massive MIMO system using Machine Learning Algorithm. Proceedings of the 2021 Sixth International Conference on Wireless Communications, Signal Processing and Networking (WiSPNET).

[19] Dao H.T., Kim S. (2020). Effective channel gain-based access point selection in Cell-Free massive MIMO systems. IEEE Access.

[20] Zhang Y., Cao H., Zhong P., Qi C., Yang L. (2018). Location-based greedy pilot assignment for cell-free massive MIMO systems. Proceedings of the 2018 IEEE 4th International Conference on Computer and Communications (ICCC).

[21] Dang X.T., Lai-Thuc T., Nguyen A.T., Vu-Huy T., Tran N.H.A., Han H.D. (2021). A Genetic Algorithm based Pilot Assignment strategy for Cell-Free massive MIMO system. Proceedings of the 2020 IEEE Eighth International Conference on Communications and Electronics (ICCE).

[22] Liu H., Zhang J., Zhang X., Kurniawan A., Juhana T., Ai B. (2019). Tabu-search-based pilot assignment for cell-free massive MIMO systems. IEEE Trans. Veh. Technol..

[23] Ding J., Kong D., Qu D. (2021). Improved Tabu-search preamble assignment in cell-free massive MIMO systems. Proceedings of the 2021 International Wireless Communications and Mobile Computing (IWCMC).

[24] Interdonato G., Ngo H.Q., Frenger P., Larsson E.G. (2019). Downlink training in cell-free massive MIMO: A blessing in disguise. IEEE Trans. Wirel. Commun..

[25] Nguyen H.V., Nguyen V.-D., Dobre O.A., Sharma S.K., Chatzinotas S., Ottersten B., Shin O.-S. (2020). On the spectral and energy efficiencies of full-duplex cell-free massive MIMO. IEEE J. Sel. Areas Commun..

[26] Buzzi S., D’Andrea C., Fresia M., Zhang Y.-P., Feng S. (2020). Pilot assignment in cell-free massive MIMO based on the Hungarian algorithm. IEEE Wirel. Commun. Lett..

[27] Al Ayidh A., Sambo Y., Imran M.A. (2022). Mitigation pilot contamination based on matching technique for uplink cell-free massive MIMO systems. Sci. Rep..

[28] Liu H., Zhang J., Jin S., Ai B. (2020). Graph coloring based pilot assignment for cell-free massive MIMO systems. IEEE Trans. Veh. Technol..

[29] Hmida W.H., Meghdadi V., Bouallegue A., Cances J.P. (2020). Graph coloring based pilot reuse among interfering users in cell-free massive MIMO. Proceedings of the 2020 IEEE International Conference on Communications Workshops (ICC Workshops).

[30] Masoumi H., Emadi M.J., Buzzi S. (2021). Coexistence of D2D communications and cell-free massive MIMO systems with low resolution ADC for improved throughput in beyond-5G networks. IEEE Trans. Commun..

[31] Lin X., Xu F., Fu J., Wang Y. (2022). Resource Allocation for TDD Cell-Free Massive MIMO Systems. Electronics.

[32] Ding Z., Adachi F., Poor H.V. (2015). The application of MIMO to non-orthogonal multiple access. IEEE Trans. Wirel. Commun..

[33] Li Y., Baduge G.A.A. (2018). NOMA-aided cell-free massive MIMO systems. IEEE Wirel. Commun. Lett..

[34] Hao Y., Xin J., Tao W., Tao S., Yu-Xiang L., Hao W. (2020). Pilot Allocation Algorithm Based on K-means Clustering in Cell-Free Massive MIMO Systems. Proceedings of the 2020 IEEE 6th International Conference on Computer and Communications (ICCC).

[35] Marzetta T.L., Yang H. (2016). Fundamentals of Massive MIMO.

[36] Cramer H. (1970). Random Variables and Probability Distributions.

[37] Tang A., Sun J.X., Gong K. (2001). Mobile propagation loss with a low base station antenna for NLOS street microcells in urban area. Proceedings of the IEEE VTS 53rd Vehicular Technology Conference, Spring 2001, Proceedings (Cat. No. 01CH37202).

